# Retinoid-mediated restoration of neurosteroidogenesis as a therapeutic target in Alzheimer’s disease

**DOI:** 10.4103/NRR.NRR-D-25-00570

**Published:** 2025-09-03

**Authors:** Pulak R. Manna

**Affiliations:** Department of Internal Medicine, School of Medicine, Texas Tech University Health Sciences Center, Lubbock, TX, USA

Regulation of neurosteroid biosynthesis is primarily mediated by the steroidogenic acute regulatory (StAR, commonly known as STARD1) protein. The StAR protein, by mobilizing the transport of intra-mitochondrial cholesterol, mediates the rate-limiting step in neurosteroid biosynthesis. The first steroid produced by the action of cytochrome P450 cholesterol side-chain cleavage enzyme (CYP11A1), at the mitochondrial inner membrane, is pregnenolone (the precursor of all neurosteroids), which is then converted to various steroids by tissue-specific enzymes. The mechanism accounting for the biosynthesis of neuro/steroids involves transcription, translation, or activation of StAR, and these processes are primarily influenced by the cyclic adenosine monophosphate (cAMP)/protein kinase A pathway, in which a plethora of signaling processes play permissible roles. An overwhelming amount of evidence indicates that gain-of-function of StAR enhances the activity of this cholesterol transporter for optimal steroid biosynthesis, and its loss-of-function strikingly decreases steroid hormones (Manna et al., 2024; Manna, 2025). The compelling evidence in the role of StAR in the regulation of steroid biosynthesis has been exemplified by numerous basic and clinical findings. Even so, dysregulation of the steroidogenic machinery, involving the hypothalamic-pituitary-thyroidal-adrenal-gonadal system, is common as life progresses from adulthood to senescence, resulting in hormonal and/or neurosteroid deficiencies. Unambiguously, neurosteroidogenesis progressively decreases during the process of aging, leading to a host of pathologies, including Alzheimer’s disease (AD), which is the most prevalent neurodegenerative disorder of geriatric populations (Manna et al., 2023a; Manna, 2025).

Whereas an early-onset, rare, and inherited familial AD accounts for 1%–2%, a late-onset and sporadic AD accounts for 70%–80% of all cases, respectively. Aging, deteriorating various neuroendocrinological activities, is the primary risk factor for widespread AD, resulting in dementia that unquestionably affects more women than men. Currently, approximately 60 million people aged 65 and older are living with AD-related dementia worldwide, which include approximately 6.9 million Americans in 2024 (Alzheimer’s Association, 2024). The pathological hallmarks of AD include brain region-specific accumulation of extracellular amyloid–beta (Aβ) plaques and intracellular neurofibrillary tangles composed of phosphorylated Tau (P-Tau), leading to cognitive decline and dementia. These events are accompanied by occluded neurosteroid biosynthesis, glial activation, microRNA dysregulation, neuroinflammation, and mitochondrial dysfunction, resulting in loss of synaptic transmission and neurons (Islam et al., 2024; Manna et al., 2024; Manna, 2025). Additionally, genetic and epigenetic alterations play significant roles in AD and AD-related dementias (ADRD), which initiates 15–20 years before the appearance of various clinical symptoms, including cognitive impairment. Despite recent understanding of AD pathogenesis, there are considerable challenges in modern-day medicine for countering this devastating disease, as there is no drug and/or therapy that can effectively treat dementia. Moreover, multifactorial etiology and disease-causing mechanisms impact AD, requiring ways to prevent, slow, ameliorate, and better manage this devastating disease that differently affects both women and men globally.

**Figure 1:**
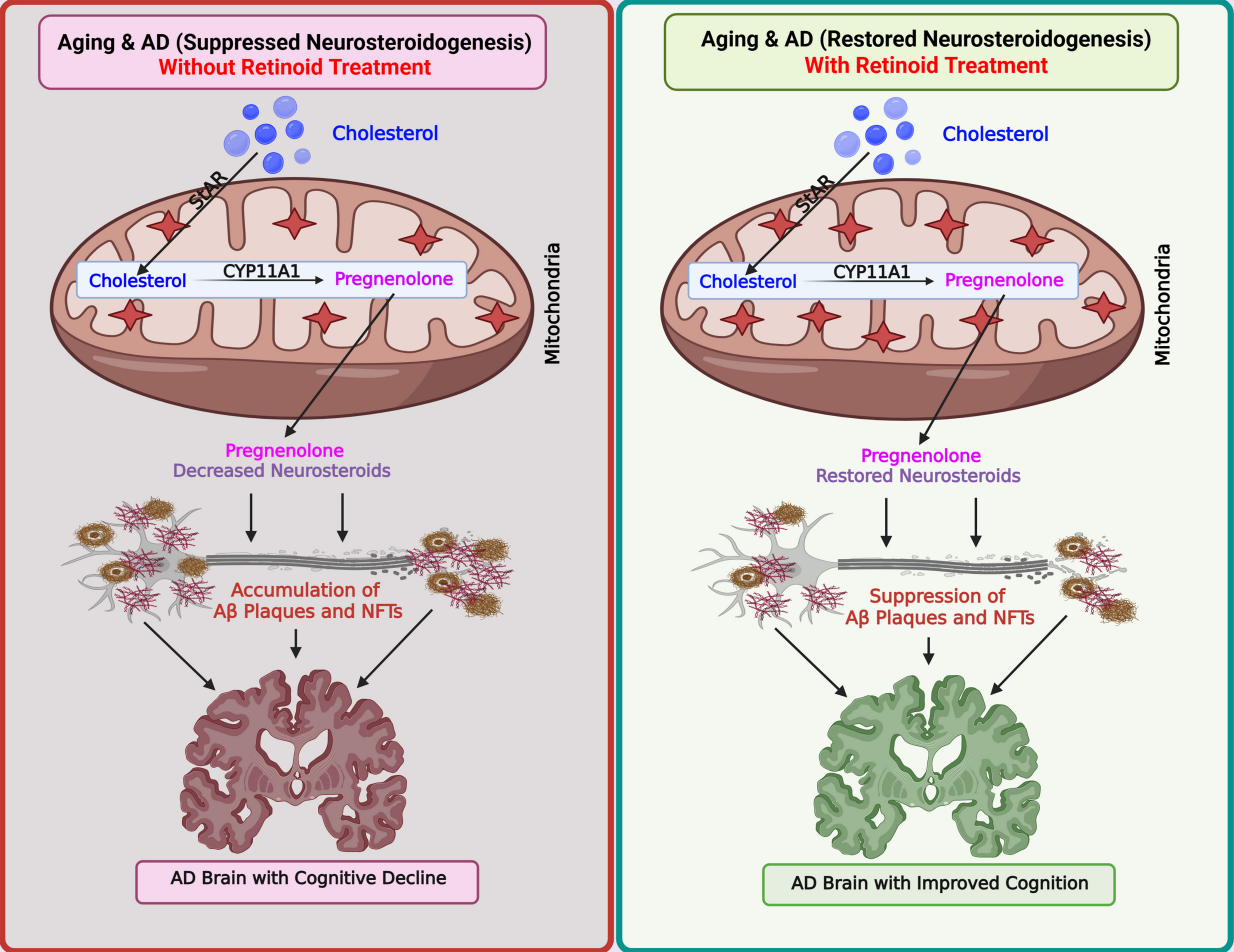
Retinoid-mediated renewal of StAR-driven neurosteroids in alleviating dementia in AD/ADRD patients. A schematic representation illustrating neuroprotective relevance of retinoid therapy in restoration of StAR-driven neurosteroids for ameliorating cognitive impairment in AD women and men. All steroids are made from cholesterol, in which the biosynthesis of neurosteroids is initiated upon mobilization of free cholesterol (cholesterol) from the outer mitochondrial membrane to the inner mitochondrial membrane. The StAR protein mediates the rate-limiting step in neurosteroid biosynthesis, i.e., the transport of intra-mitochondrial cholesterol. At the inner mitochondrial membrane, the first steroid, pregnenolone, is produced by the action of CYP11A1. Neurosteroidogenesis is suppressed in aging and/or AD/ADRD individuals, thus failing to maintain diverse neuronal activities. Disruption of neurosteroids results in accumulation of Aβ plaques and NFTs composed of P-Tau and promotes AD pathogenesis, which facilitates loss of neurons and synaptic connectivity, and differently promotes dementia in both genders (left panel). Retinoid therapy is expected to reinstate StAR-governed sex neurosteroids and simultaneously improve cognitive decline in gender-specific AD/ADRD patients. Restoration of retinoid-responsive neurosteroidogenesis results in reduction of Aβ plaques and NFTs (right panel), and these orchestrated events are fundamental for preventing, delaying, and improving cognitive decline in AD women and men. Created with BioRender.com. AD: Alzheimer’s disease; ADRD: Alzheimer’s disease-related dementias; Aβ: amyloid-beta; NFTs: neurofibrillary tangles; P-Tau: phosphorylated Tau; StAR: steroidogenic acute regulatory.

Vitamin A (retinol) and its derivatives (retinoids) play central roles in a wide variety of physiological, as well as pathological, processes. The effects of retinoids are mediated through two families of ligand-activated nuclear receptors, the retinoic acid receptor (RAR) and retinoid X receptor (RXR), each of which has three major subtypes (α, β, and γ) (Manna et al., 2024). These receptors are expressed in numerous tissues, including gonadal, adrenal, glial, and brain, suggesting that they are regulated independently and perform distinct cellular functions. Of note, dimerization of RARs-RXRs interacts with many signaling pathways and results in a large array of combinatorial actions that transduce the pleiotropic effects of retinoids. Mice lacking a number of RAR and RXR isoforms disrupt the steroidogenic machinery and affect reproductive development and function, which resembles vitamin A deficient animals. Administration of retinoids reverses not only steroidogenesis but also reproductive capacity in vitamin A deficient rats and mice, suggesting that retinoid signaling can ameliorate diverse steroid-led activities (Manna et al., 2024). We reported that retinoid signaling reverses the decline in StAR and neurosteroid levels in AD mimetic conditions and concurrently reduces diverse anomalies that promote neurodegeneration (Manna et al., 2023b, c, 2024), measures that are instrumental for restoring/improving cognitive decline and dementia for healthy aging and quality living (**Figure 1**).

**Role of steroidogenic acute regulatory in neurosteroid biosynthesis and its correlation to AD:** Neurosteroids, synthesized *de novo* from cholesterol by the action of StAR, are pivotal for diverse brain functions, including cognition, memory, mood, learning, and stress response (Manna et al., 2025). Notably, both StAR and key steroidogenic enzymes such as CYP11A1, 3β-hydroxysteroid dehydrogenase (HSD), 17α-hydroxylase and 17/20 lyase, 17β-HSD, and aromatase (CYP19A1), required for the synthesis of neurosteroids, are expressed in various brain regions, including striatum, hippocampus, amygdala, and prefrontal cortex. Since neurosteroids protect assorted neuronal activities, disruption of neurosteroidogenesis, modulating AD pathological events, is linked with gender-specific differences in AD risks. We reported that hippocampal neuronal cells overexpressing either mutant APP (K595N and M596L) or mTau (P301L), conditions mimetic to AD, suppress both StAR and pregnenolone (the precursor of all neurosteroids) levels, phenomena that are similar to AD in humans (Manna et al., 2023a, c). These data are in agreement with previous findings that demonstrated that postmortem AD brains exhibit suppression of StAR and neurosteroid levels compared with their age-matched nondemented non-AD (cognitively normal) controls, in which attenuation of StAR is inversely correlated with accumulation of Aβ and P-Tau (Manna et al., 2023a). There is increasing evidence that sex differences play a major role in gender-specific variations in AD (Lopez-Lee et al., 2024); therefore, suppression of sex neurosteroids, including progesterone, testosterone, DHEA, and estrogen, is accountable for more women developing dementia than men. In fact, deterioration of sex neurosteroids, especially progesterone and estrogen during menopause (women) and testosterone at andropause (men) respectively, promotes different AD pathogenesis (Manna et al., 2024, 2025), in which the influence of lifespans or other factors cannot be excluded. Studies have shown that dysregulation of androgen and estrogen biosynthesis has been shown to build up Aβ and P-Tau provoked anomalies in a number of sex-based AD mouse models (Manna et al., 2023b, 2024; Lopez-Lee et al., 2024). The pathophysiological significance of StAR in gender-specific differences in AD has been exemplified by analyzing RNA sequencing data for several cholesterol regulating factors and Aβ burdens in the dorsolateral prefrontal cortex brain region, which provides evidence that StAR is markedly suppressed in the brains of AD women compared with non demented control subjects, but substantially unaltered between AD and non-AD men (Manna, 2025; Manna et al., 2025). Moreover, whereas genomic expression of StAR was suppressed with cognitive diagnosis stages in the brains of AD women, it was unaffected with diverse cognitive diagnosis stages in the brains of AD men. Hence, age-related suppression of StAR-governed sex neurosteroids, promoting Aβ and P-Tau provoked neurodegenerative vulnerabilities, is a key determining event in gender-based variations of AD (**Figure 1**). It is interesting to note that overexpression of StAR in hippocampal neuronal cells, within the context of AD pathology, reverses the decline in a number of irregularities, including neurotoxicity, occluded neurosteroid biosynthesis, and mitochondrial dysfunction, and these events contribute to neurodegeneration in both women and men. As such, the regeneration of suppressed StAR-driven sex neurosteroids, involving neuronal stability, could be a novel strategy for better management of AD/ADRD/ in both sexes.

**Restoration of retinoid-mediated neurosteroidogenesis for the management of AD/AD-related dementias:** Epidemiological evidence indicates that consumption of vitamin enriched diets has numerous health benefits, as well as protective effects on many complications and diseases. Retinoids are known to exert unique modulatory and integrative roles in diverse processes, ranging from development to vision to neurodegeneration. We reported that retinoids enhance disrupted StAR and steroid levels in aged gonadal and epidermal keratinocytes, suggesting the importance of retinoid signaling in restoration of the steroidogenic response (Manna et al., 2015, 2023b, c). Moreover, age-related deterioration of StAR expression and activity, thus, neurosteroids, promoting neuronal imbalance, is associated with a greater risk of developing dementia in women than men. Accordingly, restoration of StAR-driven sex neurosteroids, reducing Aβ and P-Tau provoked pathologies, has the potential for preventing and managing AD-related dementia in both women and men. Nonetheless, we have shown that atRA, 9-cis RA, and selective analogs with affinities to both RAR and RXR are capable of enhancing StAR expression and neurosteroid biosynthesis in hippocampal neuronal cells (Manna et al., 2023b, c, 2024). The mechanism accounting for retinoid-mediated induction of the neurosteroidogenic machinery involves the interaction with an overlapping RAR/RXR-LXR (liver X receptor) heterodimeric motif in the StAR promoter. Concomitantly, the functional interaction and/or cooperation between retinoid and LXR analogs in synergistic response to steroidogenesis have been demonstrated in gonadal and neuronal cells (Manna et al., 2015, 2023b). Besides, marked increases in StAR, gonadal-, and neuro-steroid levels in response to retinoids are evident by activation of the cAMP/protein kinase A pathway, signifying involvement of StAR phosphorylation-independent and -dependent events, respectively, in retinoid responsiveness (Manna et al., 2023b, c). Notably, retinoids were found to restore suppressed StAR expression and neurosteroid biosynthesis impelled by Aβ and P-Tau accumulation in hippocampal neuronal cells. These findings emphasize that retinoid-mediated restoration of neurosteroidogenesis, impacting AD pathology, is a key event improving cognitive function in AD patients. Accordingly, retinoids, by elevating the neurosteroidogenic machinery, can reinstate diverse neurodegenerative events, including neurotoxicity, mitochondrial dysfunction, and epigenetic dysregulation. Along these lines, our findings provide novel insights into the mechanistic events elucidating that an abundance of mitochondrial StAR-governed neurosteroids reduces a number of Aβ and P-Tau provoked anomalies (Manna et al., 2023b, c), measures that are accountable for preventing and/or ameliorating AD-related dementia (**Figure 1**). It is plausible that an increase in neuronal StAR expression and activity facilitates its cholesterol transporting capacity for enhanced biosynthesis of sex neurosteroids, which can be considered as a novel approach for the management of dementia in both AD women and men. Hence, retinoid-linked therapeutic compounds such as Am80, bexarotene, and atRA used frequently for treating dementia in AD patients, appear to enhance the biosynthesis of neurosteroids (Behl et al., 2022; Manna et al., 2023b, 2024). Additionally, an antibody to Aβ, aducanumab, which has been approved by the U.S. FDA for the improvement of cognitive decline and dementia, may enhance StAR and neurosteroid levels that are impaired in AD/ADRD. Therefore, retinoid therapy, by upregulating the neurosteroidogenic machinery, is capable of alleviating cognitive impairments in both AD women and men, benefiting the health and quality of life of geriatric populations. Collectively, retinoid-mediated regeneration of StAR-governed neuro/steroids is expected to improve AD and other age-related irregularities, including (i) reinstating/alleviating hormonal balance and immunosuppression, (ii) reestablishing neurosteroid stability, (iii) reversing defects connected with vitamin A deficiency, (iv) ameliorating vision, muscular, and bone health, (v) restoring abnormalities connected with hypogonadism, (vi) returning epidermal homeostasis for skin rejuvenation, and (vii) regressing atherosclerotic cardiovascular disease. All of these restorative processes are influential for preventing, delaying, or ameliorating cognitive decline and dementia, benefiting millions of AD patients. Moreover, restoration of StAR-governed sex neurosteroids is neuroprotective in mitigating AD/ADRD for elderly women and men to live longer in good health.

**Concluding remarks and future directions:** Aging, involving endocrino-immuno-senescence, promotes AD-related dementia in which accumulation of Aβ plaques and P-Tau plays an indispensable role. Postmortem AD brains exhibit suppression of StAR, concomitant with neurosteroids, compared with their age-matched nondemented subjects, and these events are more pronounced in women than men (Manna et al., 2024, 2025), underscoring that age-related progressive disruption of neurosteroidogenesis is a key event for developing AD. Thus, StAR-governed suppression of sex neurosteroids, enabling menopause *vs*. andropause, differently influences gender-specific AD pathology. Intrinsically, the protective function of sex neurosteroids in AD involves renewal of neuronal stability for maintaining susceptibilities that trigger neurodegeneration in both genders. It is noteworthy that retinoids, by binding and/or interacting with the RAR/RXR-LRX motif in the StAR promoter, augment the transport of intra-mitochondrial cholesterol for additional synthesis of sex neurosteroids (Manna et al., 2024), which may improve cognitive decline in AD/ADRD patients. Nonetheless, an increase in StAR, thus, neurosteroids, either by its overexpression or retinoids, in AD mimetic conditions, reverses the decline in a number of Aβ and P-Tau provoked neurodegenerative susceptibilities. Moreover, retinoid therapy, by sustaining hormonal/neuronal stability and immunomodulation, can be a novel therapeutic target not only for AD/ADRD, but also for a number of age-associated complications/diseases. Future studies, elucidating a comprehensive understanding of retinoid-mediated upregulation of StAR-governed sex neurosteroids, could help evolve new drug(s) that can be combined with any traditional therapies for managing dementia. Accordingly, the results obtained will lay the groundwork for future clinical trials with retinoids and/or other compounds for developing gender-based personalized therapies for preventing, delaying, or ameliorating dementia in mild cognitive impairment and AD/ADRD women and men for healthy aging.

*This work was supported in part by funding from the Department of Internal Medicine and The CH Foundation (to PRM).*
